# Post-translational modifications of PRC2: signals directing its activity

**DOI:** 10.1186/s13072-020-00369-1

**Published:** 2020-10-31

**Authors:** Yiqi Yang, Gang Li

**Affiliations:** 1 Faculty of Health Sciences, University of Macau, Macau, China; 2 Cancer Centre, Faculty of Health Sciences, University of Macau, Macau, China; 3 Centre of Reproduction, Development and Aging, Institute of Translational Medicine, Faculty of Health Sciences, University of Macau, Macau, China

**Keywords:** PRC2, Cis chromatin features, CGIs, Facultative subunits, PTMs, Methylation, Phosphorylation, Ubiquitination

## Abstract

Polycomb repressive complex 2 (PRC2) is a chromatin-modifying enzyme that catalyses the methylation of histone H3 at lysine 27 (H3K27me1/2/3). This complex maintains gene transcriptional repression and plays an essential role in the maintenance of cellular identity as well as normal organismal development. The activity of PRC2, including its genomic targeting and catalytic activity, is controlled by various signals. Recent studies have revealed that these signals involve cis chromatin features, PRC2 facultative subunits and post-translational modifications (PTMs) of PRC2 subunits. Overall, these findings have provided insight into the biochemical signals directing PRC2 function, although many mysteries remain.

## Background

Epigenetics can be defined as “a stably heritable phenotype resulting from changes in a chromosome without alterations in the DNA sequence [1].” Practically, the term "Epigenetics" refers to DNA and chromatin modifications that persist during cell division [1, 2]. Epigenetic regulation is essential for cell fate decisions and cellular functions. With minor exceptions, most development and differentiation processes are triggered and maintained through epigenetic mechanisms [3]. Polycomb group (PcG) proteins are epigenetic regulators that function by modifying chromatin.

The PcG proteins were originally found in *Drosophila melanogaster* over 70 years ago [4]. Later, they were determined as negative regulators of homeotic (Hox) genes, a gene family controlling thoracic and abdominal development [5]. Since then, an increasing number of PcG proteins have been identified, most of which are conserved across organisms, ranging from yeast [6], filamentous fungi [7], plants [8] to animals [9], and they even have been found in various unicellular eukaryotes (for a review, see [10]). Multiple PcG proteins can coordinate and assemble into large multimeric protein complexes with distinct functions. In mammals, they form two major complexes, namely, Polycomb Repressive Complex 1 (PRC1) and 2 (PRC2). PRC1 catalyses the monoubiquitylation of histone H2A at lysine 119 (H2AK119ub1) [11, 12], which is required for the Polycomb-mediated transcriptional repression [13, 14]. In contrast, PRC2 catalyses the methylation of histone H3 at lysine 27 (H3K27me1/2/3), and histone H3K27 dimethylation and trimethylation (H3K27me2/3) are closely related to silent genomic regions [15–19].

**Fig. 1:**
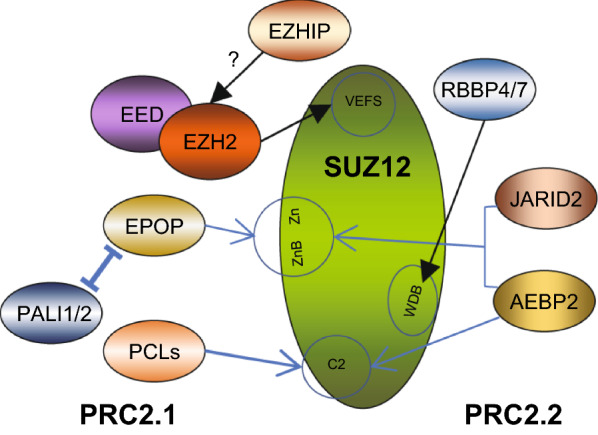
Schematic illustration of the assembly of the PRC2 holoenzyme. EZH2 associates with EED and EZHIP. SUZ12 functions as a structural platform that orchestrates distinct sets of facultative subunits to form PRC2.1 and PRC2.2. Circles and arrows indicate the domains of SUZ12 interacting with core PRC2 and facultative subunits. ZnB, zinc finger-binding; WDB1/2, WD-40 binding domain1/2; Zn, Zn finger region; VEFS, VRN2-EMF2-FIS2-SUZ12 domain

Mammalian PRC2 contains three core subunits: EZH1/2, EED and SUZ12. The catalytic activity of PRC2 relies on the SET domain of EZH1/2 [15, 17–19]; however, EZH1/2 exhibits no detectable histone methyltransferase (HMTase) activity by itself, and for which both of EED and SUZ12 are indispensable [20–22]. Indeed, EED binds to H3K27me3 and allosterically stimulates the enzymatic activity of PRC2 [23], while SUZ12 functions as a structural platform that stabilizes the PRC2 holoenzyme and defines distinct PRC2 subcomplexes by associating with RBBP4/7 and other sub-stoichiometric partners (facultative subunits) (Fig. 1) [24–26]. Proteomic analyses have identified two alternative subtypes of PRC2, namely, PRC2.1 and PRC2.2 [27]. PRC2.1 includes one of the Polycomb-like (PCL) proteins–PHF1, MTF2 or PHF19, and either PALI1/2 or EPOP, while PRC2.2 contains AEBP2 and JARID2 [27–29]. In addition, recent studies have identified a tissue-specific PRC2 component EZHIP, which can associate with EZH2 [30–33]. These facultative subunits are not strictly necessary for core PRC2 formation, yet their presence affects the PRC2 recruitment and catalytic activity (discussed in detail below).

The crucial role of PRC2 in development is highlighted by the early embryonic lethality of mice lacking the PRC2 core subunits [34–36]. In humans, germline monoallelic mutations in PRC2 subunits may cause multisystem genetic disorders, such as overgrowth-intellectual disability (OGID) syndromes [37–39], while mutation or dysregulation of PRC2 subunits is frequently observed in multiple cancers and diseases, including diffuse large B-cell lymphoma (DLBCL) [40], T-cell acute lymphoblastic leukaemia (T-ALL) [41], myelodysplastic syndrome/myeloproliferative neoplasm (MDS/MPN) [42], glioblastoma multiforme (GBM) [43, 44] and melanoma [43], suggesting a pathogenic or carcinogenic role of aberrant forms of PRC2. In recent years, several small-molecule inhibitors targeting EZH2 in cancer therapy have entered clinical trials [45, 46], suggesting that pharmacological intervention may be possible in human diseases in which PRC2 systems are frequently perturbed. Therefore, a comprehensive understanding of the mechanisms controlling PRC2 activity will contribute to a better understanding of human diseases with an aberrant Polycomb system and thus provide inspiration for the development of new therapeutic strategies targeting PRC2.

Here, we discuss the latest advances regarding the mechanisms that govern PRC2 activity. These include cis chromatin features, PRC2 facultative subunits and post-translational modifications (PTMs) that occur in PRC2 subunits. We conclude by discussing the unresolved issues and future directions related to PRC2 function.

## Regulation of PRC2 recruitment and catalytic activity: current status

### Cis chromatin features

#### Specific characteristics of PRC2-binding regions

**Fig. 2:**
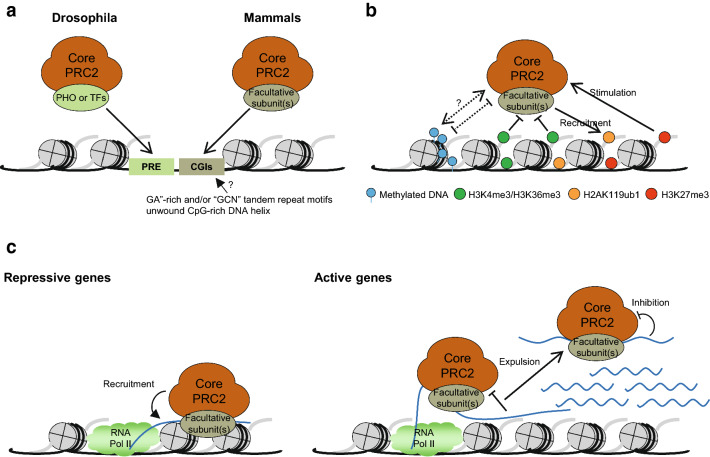
Cis chromatin features regulating PRC2 enzymatic activity or genomic targeting. **a** Features of PRC2-binding regions. In *Drosophila*, PREs were identified as DNA elements that recruit PRC2 via interaction with other PcG proteins (i.e., PHO) or TFs. In mammals, both the DNA motif sequence and conformation in CpG islands (CGIs) regulate PRC2 recruitment. **b** DNA methylation and histone modifications affect PRC2 activity. DNA methylation and PRC2 can be mutually exclusive or can coexist, depending on cell context, suggesting that unknown factors remain to be determined (left). Histone modifications H3K4me3 and H3K36me3 constrain PRC2 activity (middle), while H3K27me3 and H2AK119ub1 stimulate PRC2 catalytic activity or direct its recruitment, respectively (right). **c** Nascent RNAs regulating PRC2 recruitment depend on transcriptional status. PRC2 interacts promiscuously with multitudinous RNAs. For the repressed genes (left), RNA is transcribed at a very low rate, and the majority of the RNA remains attached to chromatin. Thus, PRC2 bound to the RNA is in very close proximity to the chromatin, allowing PRC2 to slowly deposit H3K27me3 despite low activity. Although binding to RNA antagonizes the allosteric activation of PRC2, these inhibitory effects gradually decrease with the accumulation of H3K27me3, ultimately establishing stable PRC2-mediated gene repression. However, in the active genes that are largely free of PRC2 (right), RNA is transcribed at a very high rate, and most of the RNA is freed from chromatin. Hence, any PRC2 bound to RNA is also consequently removed with inhibited activity, and new RNA can be transcribed continuously, eventually expelling PRC2 from the chromosome

Early works in *Drosophila melanogaster* identified the polycomb response elements (PREs) as DNA regulatory elements that facilitate the recruitment of PRC2 to chromatin (Fig. 2a) [47]. Several PcG proteins, such as PHO and its homologue PHO-like (PHOL), or transcription factors (TFs), such as the Dorsal switch protein (DSP1), GAGA factor (GAF) and Pipsqueak (Psq), can associate with their cognate DNA motifs in PREs [48, 49]. Thus, the initial PRC2 can be recruited to chromatin through transient interaction with these PcG proteins or TFs (Fig. 2a) [49, 50]. The PREs in flies are reasonably well characterized, while their mammalian counterparts seem highly elusive and most TFs participating in *Drosophila* PRC2 recruitment either are not conserved in mammals or do not function in mammalian PRC2 recruitment, implying that mammals have different recruitment mechanisms.

The most common feature of the mammalian PRC2-binding region is the presence of CpG islands (CGIs) (Fig. 2a). In mouse embryonic stem cells (mESCs) and human embryonic stem cells (hESCs), more than 90% of the PRC2-enriched regions closely correspond to CGIs or CpG-rich regions, which lack DNA methylation and are adjacent to the transcription start site (TSS) of the promoter in the silenced genes, suggesting that CGIs may contribute to PRC2 recruitment in mammals [51–54]. In support of this notion, integrating an artificial unmethylated CGI-like DNA into non-transcribed genomic regions is sufficient to recruit PRC2 [55, 56], while removing the activating motifs or inhibiting transcription can ectopically recruit PRC2 to the CpG-rich promoter of the active genes [56–58]. In addition, several PRC2 facultative subunits, such as JARID2 and PCLs, also exhibit binding preferences for CpG-rich sequences [59–61]. Therefore, these CGIs in mammals are somewhat similar to PREs in *Drosophila*, which contain DNA motifs to direct the recruitment of PRC2.

Most mammalian gene promoters contain CGIs, but only a minority are PRC2-positive, indicating that these cis-elements should have their own characteristics (Fig. 2a). Indeed, DNA motif analysis of the PRC2-binding regions in mESCs demonstrated that PRC2 is initially recruited to the “nucleation site” enriched for “GA”-rich and/or “GCN” tandem repeat motifs in the CGI promoter [62]. These sequence motifs are unique compared with other parts of the CGIs, suggesting that they can be recognized by PRC2 and contribute to its recruitment [62]. In addition, another study proposed that both the unmethylated DNA sequence and the DNA helical shape in the CGIs are critical for PRC2 binding [63]. Indeed, the unmethylated GCG trinucleotide motif showing an unwound DNA helix (compared to canonical B-DNA) can specifically recruit PRC2-MTF2 and nucleate the Polycomb domain, while the target GCG trinucleotide motif without the preferred DNA shape or with DNA methylation cannot direct PRC2-MTF2 binding to DNA [63]. Collectively, these observations suggest that both the DNA motif sequence and its conformation are critical for initial mammalian PRC2 recruitment.

#### DNA methylation: mutually exclusive or coexisting with PRC2?

High-density DNA methylation seems to be mutually exclusive with PRC2, since most of the CGIs or CG-rich regions occupied by PRC2 are hypomethylated, and in multiple confirmed studies, the removal of DNA methylation leads to the ectopic accumulation of PRC2 and H3K27me3 in the previously methylated DNA regions [55, 64–66]. However, a recent report revealed that DNA methylation does not inhibit the enzymatic activity of PRC2 in vitro, and both PRC1 and PRC2 can be artificially recruited to naturally hypermethylated DNA regions, suggesting that DNA methylation is not directly antagonistic to PRC2 in vivo [67]. Moreover, the PRC2 facultative subunit AEBP2 can recognize and specifically interact with mCpG dinucleotides through its conserved C2H2 zinc finger domain, thereby preferentially mediating PRC2 binding to methylated DNA in vitro [68]. Furthermore, DNA methylation and H3K27me3 coexist during mouse X chromosome inactivation and upon mouse fibroblast immortalization or tumorigenic transformation [69–71]. In addition, the deposition of H3K27me3 in human promyelocytic cells relies on DNA methylation [71]. These data suggest that DNA methylation and PRC2 coexist in several contexts. Clearly, additional insights are required to determine the cause of these phenomena, especially whether AEBP2 or any unknown factors are involved in this process. Nevertheless, these observations suggest that PRC2 might not have any preference for DNA methylation in the chromatin region, while the mutual exclusion of DNA methylation and H3K27me3 in the genome is probably dependent on context with unknown factors.

#### Histone modifications affecting PRC2 enzymatic activity or genomic targeting

Histone modifications in the chromatin region also affect PRC2 binding or catalytic activity, either stimulation or blocking (Fig. 2b). One example is PRC2’s own catalytic product, H3K27me3, which was shown to interact with the aromatic cage of EED, leading to the allosteric activation of PRC2 and further deposition of H3K27me3 [23, 72]. However, this EED-H3K27me3 interaction is dispensable for PRC2 recruitment, since H3K27me3 has little effect on the overall PRC2 nucleosome binding [68], and loss of EED [73] or mutations in the EED aromatic cage [62] will disrupt this interaction but will not abolish PRC2 recruitment [62, 73]. In addition, the PRC1 catalytic product, H2AK119u1, can interact with JARID2 to facilitate PRC2 recruitment and H3K27me3 deposition [67, 74]. These observations, along with the hierarchical model that H3K27me3 can associate with CBX and direct PRC1 recruitment [50], suggest that PRC1 and PRC2 may contribute synergistically to enhance their genomic targeting and promote the formation of the Polycomb chromatin domain.

In addition to the catalytic products of the PRCs themselves, H3K4me3 and H3K36me3, which are catalysed by MLL/COMPASS family proteins and methyltransferase HYPB/Setd2, respectively, can also regulate PRC2 activity [75, 76]. Indeed, the in vitro enzymatic activity of PRC2 is inhibited by pre-existing H3K4me3/H3K36me3 in the same histone H3 polypeptides or nucleosomes [77–79]. However, this inhibition might be an intrinsic property of PRC2 that is independent of its recruitment, as the in vitro assays showed that H3K4me3 does not affect the overall PRC2 nucleosome binding [68, 77], while the PRC2 facultative subunits PCLs can preferentially bind to H3K36me3, inhibiting PRC2 activity but leaving the chromatin binding unperturbed [59, 79, 80]. Moreover, it has been reported that PHF19 binds H3K36me3 and interacts with H3K36me3 demethylase NO66 [81] and KDM2b [82], whereas PRC2 interacts with H3K4me3 demethylase RBP2 (JARID1A/KDM5A) [83], respectively, to facilitate the removal of H3K36me3 and H3K4me3 and the deposition of H3K27me3. These observations may provide a potential mechanism for the transition of the active transcription state to the Polycomb-repressed state; however, these results require further confirmation.

Overall, the evidence presented so far indicates that histone modifications are likely to affect the deposition of H3K27me3 by affecting PRC2 catalytic activity or binding preference. Although none of them can fully explain the specific targeting of PRC2 to CGIs, the catalytic products of the PRCs themselves contribute to H3K27me3 deposition. In addition, the repression of PRC2 by transcription-related histone modifications allows PRC2 activity or H3K27me3 to be excluded from transcribed regions, while the participation of PRC2 facultative subunits and the demethylase or deacetylases in these histone modifications provides a reasonable explanation for the transition of genes from an active transcriptional state to a Polycomb-repressed state.

#### Nascent RNAs regulating PRC2 recruitment depended on transcriptional status

Genome-wide RIP-seq analysis has identified various RNAs interacting with PRC2 in vivo, indicating that RNA may regulate PRC2 activity (Fig. 2c) [84, 85]. Indeed, short abortive RNAs transcribed at low levels from the repressed genes can interact with PRC2 through its stem–loop structure, which may tether PRC2 to target gene promoters to maintain gene repression or inhibit aberrant transcription [86]. However, nascent RNA transcribed from a highly expressed gene locus can also interact with PRC2, which might competitively inhibit the interaction between chromatin and PRC2 [87] or serve as a decoy to limit PRC2 binding to chromatin [68, 84]. Notably, the main PRC2–RNA binding regions include the N-terminus of the CXC domain of EZH2 and the helical structure between its SEB and EBD domain, which are critical for both PRC2–DNA/nucleosome interaction and the allosteric activation of PRC2 [88, 89]. Thus, RNAs can facilitate or impede PRC2–chromatin interactions while inhibiting the catalytic activity of PRC2.

In a seeming paradox, the intrinsic function of PRC2 is to maintain gene repression, but interaction with RNA may inhibit the enzymatic activity of PRC2. In addition, it is unclear how the participation of RNA in both the recruitment and the eviction of PRC2 is balanced. We speculate that this balance depends on the rate of RNA release from the genomic locus. At the repressed Polycomb target genes, where there is a lack of transcriptional machinery and active histone modifications, RNA is transcribed at a very low rate, and most of the RNA remains attached to chromatin. Hence, once PRC2 binds to RNA, both are in very close proximity to chromatin, which allows PRC2 to slowly deposit H3K27me3 despite low activity. Although binding to RNA antagonizes the allosteric activation of PRC2, these inhibitory effects gradually decrease with the accumulation of H3K27me3. Moreover, PRC2–PCL holo complex dimerizes intrinsically [90], which potentially promotes PRC2 accumulation, ultimately establishing stable PRC2-mediated gene repression. However, in the active genes that are independent of PRC2, RNA is transcribed at a very high rate, and the majority of the RNA is released from the chromatin. Thus, any PRC2 bound to RNA is subsequently removed with inhibited activity, and the new RNA can be continuously transcribed, eventually expelling PRC2 from the genomic locus.

### Facultative subunits of PRC2

#### PCLs and JARID2: two facultative subunits regulating PRC2 recruitment

**Fig. 3:**
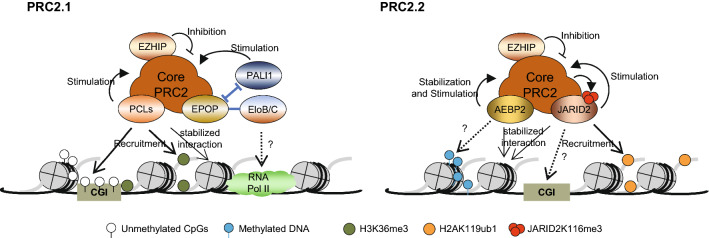
Facultative subunits regulating PRC2 activity. (Left) Subunits in PRC2.1 regulate recruitment and activity. All PCLs can recruit PRC2.1 to unmethylated CGI and associate with H3K36me3 for specific targeting. In addition, PHF1 can extend the residence time of PRC2 in chromatin and stimulate its catalytic activity. PALI1 can stimulate the catalytic activity of PRC2.1, whereas its mutually exclusive subunit EPOP is likely to associate with EloB/C to maintain low levels of transcription. (Right) Subunits in PRC2.2 regulate recruitment and activity. Both AEBP2 and JARID2 can stimulate PRC2.2 activity and increase its binding affinity to nucleosomes. AEBP2 is a stabilizing subunit of PRC2.2 and can bind to methylated DNA in vitro, but whether this binding specificity affects PRC2 recruitment remains uncertain. JARID2 can facilitate the recruitment of PRC2.2 through interaction with H2AK119ub. In addition, JARID2 recognizes and binds to GC-rich DNA in vitro, but the function of this preference remains to be determined. Finally, JARID2 can also be methylated by PRC2, which may in turn allosterically activate the enzymatic activity of PRC2. EZHIP exists in both PRC2.1 and PRC2.2 and functions as a robust inhibitor of PRC2 activity

The PCL homologues (PHF1, MTF2, and PHF19) are part of PRC2.1 that modulate the specific targeting of PRC2 in chromatin (Fig. 3). In support of this notion, ablation of the individual PCL proteins modestly affected the recruitment of PRC2, while triple knockout of PCL paralogues led to a dramatic reduction in PRC2 occupation and H3K27me3 deposition [63, 91–95]. Indeed, PCL proteins can facilitate PRC2 binding to unmethylated CG-rich DNA sequences through their N-terminal extended homologous (EH) domains, which indicates a direct role for PCLs in linking CGIs and PRC2 [59, 63]. In addition, all three PCLs have been shown to bind H3K36me3 peptide in vitro due to their tudor domain [59, 82, 96], which provide a potential explanation for the transition of the active transcription state to the Polycomb-repressed state. However, the enrichments for H3K27me3 and H3K36me3 are mutually exclusive in vivo, and the deposition of H3K27me3 is inhibited by H3K36me3-bearing nucleosomes [79]; hence, there is a lack of definitive in vivo evidence that PCLs mediate the targeting of PRC2 through H3K36me3. Besides, PHF1 can also enhance the enzymatic activity of PRC2 both in vivo and in vitro [97], and PRC2 with PHF1 exhibits extended residence time on DNA and chromatin compared to the core PRC2 alone [98]. Together, these studies indicate that PCL proteins play an essential role in the recruitment of PRC2 and might also contribute to stabilizing PRC2–chromatin association and stimulating PRC2 activity.

JARID2 is a PRC2.2 component that can both mediate PRC2 recruitment and stimulate its catalytic activity (Fig. 3). The basis of PRC2 stimulation by JARID2 might partly rely on its N-terminal nucleosome-binding domain, which can stabilize the interaction with chromatin [99]. Moreover, JARID2 can be methylated by PRC2 at lysine residue 116 (JARID2-K116me), and similar to H3K27me3, JARID2-K116me3 can be recognized by the aromatic cage of EED, which may in turn allosterically activate the enzymatic activity of PRC2 [100, 101]. In addition to PRC2 stimulation, JARID2 also participates in PRC2.2 recruitment. Indeed, JARID2 also exhibits DNA binding affinity through its C-terminal AT-rich interaction domain (ARID) and Zn (zinc finger) domains, with a slight preference for GC-rich sequences [60, 102] that is consistent with the general features of the PRC2 binding site [56, 61, 64]. Although it remains to be determined whether this CpG preference exerted through DNA-binding properties has a functional effect on the recruitment of PRC2 to the CGIs in cells, JARID2 does significantly colocalize with PRC2 in the genome, while the deletion of JARID2 in mESCs decreases both PRC2 occupation and H3K27me3 deposition [60, 61, 91, 95, 103]. In addition, recent studies have shown that JARID2 can facilitate the interaction between PRC2 and H2AK119u1 via its ubiquitin-interacting motif (UIM) in vitro and in cells, leading to the deposition of H3K27me3 in chromatin, which suggests that JARID2 might synergize with PRC1 to mediate the specific targeting of PRC2 [67, 74]. Thus, JARID2 functions as a PRC2 recruiter and modulator to regulate the PRC2–chromatin binding pattern and catalytic activity.

#### Other facultative subunits regulating PRC2 activity

EPOP is a mammalian-specific PRC2.1 component whose function has been unravelled [104–106]. Compared to other PRC2 subunits, EPOP lacks any known functional domains and may be mostly unstructured other than the N- and C-termini [107]. Despite stimulating PRC2 catalytic activity in vitro [104], EPOP inhibits PRC2 in cells [106, 107] and occupies both active and repressed genes [108]. Indeed, immunoprecipitation and mass spectrometry analysis have identified EPOP as a scaffold protein linking PRC2 and EloB/C [106, 108]. Notably, EloB/C is a stable heterodimer and part of the canonical Elongin complex, which can stimulate the elongation activity of RNA polymerase II [109]. Thus, this EloB/C-PRC2 interaction suggests that EPOP might contribute to the transcription of PRC2-targeting genes. In support of this notion, the ablation of EPOP disturbs the EloB/C-PRC2 interaction, leading to a mild decrease in the expression of these genes, with a slight increase in PRC2 and H3K27me3 occupancy [106]. Thus, it is likely that EPOP may constrain PRC2 function to maintain repressive genes at a basal level of transcription.

PALI1 is a vertebrate-specific PRC2.1 facultative subunit that appears mutually exclusive with EPOP [105, 110]. Notably, PALI1 is a fusion protein that arises from alternative splicing of transcripts originating from Ligand dependent nuclear receptor corepressor (LCOR) gene loci, which encompass the LCOR and C10ORF12 proteins, previously linked with PRC2.1 [110]. Similar to PALI1, another paralogue originating from the Ligand dependent nuclear receptor corepressor like (LCORL) gene loci, namely, PALI2, can also interact with PRC2.1 [110]. A previous study relied on the luciferase reporter system and showed that both LCOR and C10ORF12 proteins can mediate transcriptional repression and induce H3K27me3 deposition, suggesting that this protein can enhance PRC2 function by assembling into the PRC2.1 subcomplex [27]. Consistently, PALI1 stimulates PRC2 catalytic activity in vitro; however, the excision of PALI1 in mESCs causes only a slight decrease in the H3K27me2/3 level in a subset of PRC2 target genes [110], which may be due to the relatively low stoichiometry of PALI1 compared to other PRC2.1 components [111, 112]. Therefore, PALI1 might function as a positive modulator to fine-tune PRC2 activity.

AEBP2 is another PRC2.2 facultative subunit that can enhance PRC2 activity (Fig. 3). Indeed, AEBP2 can stimulate PRC2 catalytic activity in vitro, while the addition of JARID2 may further synergistically increase the enzymatic activity of PRC2 [20, 99, 113]. The detailed mechanism by which AEBP2 stimulates PRC2 activity remains unclear; however, AEBP2 can stabilize PRC2 through association with the SET domain of EZH2, SUZ12 and RBAP46/48, which may in turn keep PRC2 in an enzymatically active conformation [24]. In addition, AEBP2 can improve the binding capacity and stability of PRC2 to nucleosomes [114], while ablation of the SUZ12 C2 domain (domain for AEBP2 and PCL binding) will reduce the PRC2 residence time in chromatin [115]. Moreover, AEBP2 can recognize and bind to methylated DNA through its C2H2 zinc finger domain, which might affect the binding preference of PRC2 in vitro [68, 116]; however, it remains to be verified whether this binding specificity plays a role in PRC2 stimulation or recruitment since high levels of DNA methylation rarely coexist with H3K27me3 at the same loci in mammalian genomes [117–119]. In contrast to its stimulating role in vitro, AEBP2 appears to constrain PRC2 activity in cells, as disruption of AEBP2 in mESCs results in slightly elevated levels of PRC2 and H3K27me3 enrichment at their target sites [120]. It remains unclear how AEBP2 could limit PRC2 activity in vivo. One potential hypothesis might be that AEBP2 is centrally localized in PRC2.2 via association with several core subunits, and AEBP2 knockout disrupts this balance and enables rearrangement of the subunit composition of PRC2, resulting in PRC2.1/PRC2.2 hybrid complexes containing both PALs and JARID2 [24, 120, 121]. It is possible that these PRC2.1/PRC2.2 hybrid complexes could enhance PRC2 recruitment or catalytic activity [120], since the stimulation of PRC2.1 catalytic activity by MTF2 and EPOP is drastically increased with the addition of JARID2 in vitro [104]. Together, these observations suggest that AEBP2 might play a role in controlling the stability and subunit composition of the PRC2 subcomplexes and thus regulate the catalytic activity of PRC2. AEBP2 might also contribute to affecting the DNA preference of PRC2; however, all these processes remain to be further studied.

EZHIP is a tissue-specific PRC2 facultative subunit that exists in both PRC2.1 and PRC2.2 [30–32]. EZHIP does not affect the recruitment of PRC2 to chromatin [30]; however, it inhibits PRC2 activity both in vitro and in vivo [30–33]. In support of this notion, the expression of EZHIP transgenes leads to a genome-wide reduction in H3K27me3 [32], while the removal of EZHIP causes ectopic enrichment of H3K27me3 in chromatin, but has little effect on SUZ12 deposition [30]. The mechanism by which EZHIP inhibits PRC2 activity is quite controversial: several studies have reported that the C-terminus of the EZHIP contains a highly conserved “K27M-like” sequence that binds to the SET domain of EZH2, and thus blocking the PRC2 catalytic activity [32, 33], while another study proposed that EZHIP might reduce the interaction between the core subunit and facultative subunits (e.g., AEBP2 and JARID2) to limit their stimulation of PRC2 enzymatic activity [30]. Although more work is needed to resolve the above disputes, these data indicate that EZHIP may function as a robust inhibitor of PRC2 activity.

## PTMs fine-tune PRC2 in multiple processes

Accumulating evidence indicates that post-translational modifications (PTMs) of epigenetic regulators comprise the signals mediating the establishment of epigenetic landscapes. Likewise, PCR2 subunits are modified by various PTMs, including methylation, phosphorylation, acetylation, ubiquitination, SUMOylation and O-GlcNAcylation. Indeed, a search of PhosphoSitePlus^®^ (PSP, https://www.phosphosite.org/) [122], a knowledge base of curated information on PTMs of proteins, revealed 418 PTM sites in human PRC2, ranging from 6 to 70 for each subunit (See Additional file 1). The functions of a fraction of these PTMs have been revealed, particularly the PTMs of EZH2 (Table 1).

**Table 1:** PTMs of PRC2 subunits and their functions

Type of PTM	Subunit	Condition	Modifying enzymes	Sites (human residue)	Function	References
Methylation	EZH2		PRC2	K510, K514 and K515	Facilitates PRC2 access to histone H3K27 substrate, critical for H3K27me3 catalysis	[113, 125]
	SUZ12		PRC2	Unknown	Unknown	[113]
	JARID2		PRC2	K116	Allosteric activation of PRC2	[100]
Phosphorylation	EZH2	IGF-induced	PKB/Akt	S21	Disturbs PRC2 interaction with histone H3, inhibits PRC2 catalytic activity, decreases H3K27me3 level	[126]
			CDK1	T345 (mouse)	Promotes PRC2 interaction with HOTAIR, recruitment, promotes ubiquitin-mediated degradation	[128, 134]
		TNFα	p38α	T372 (mouse)	Enhances PRC2 interaction with YY1, recruitment	[127]
			CDK2	T416	Enhances PRC2 interaction with NIPP1, recruitment	[129, 130]
			AMPK	T311	Disrupts binding to other PRC2 components, inhibits PRC2 catalytic activity	[131]
			JAK3	Y244	Disrupts binding to other PRC2 components, inhibits PRC2 catalytic activity	[132]
			CDK1	T487 (mouse)	Disrupts binding to other PRC2 components, decreases H3K27me3 level, promotes ubiquitin-mediated degradation	[133, 134]
			CDK5	T261	Promotes ubiquitin-mediated degradation	[136]
			JAK2	Y641	Promotes ubiquitin-mediated degradation	[135]
	SUZ12		PLK1	S539, S541 and S546	Disrupts binding to other PRC2 components, promotes ubiquitin-mediated degradation	[137]
	ESC (*Drosophila*)		CK1/CK2	N-terminus	EED homodimerization and larger PRC2 complex stability	[138]
Acetylation	EZH2		PCAF	K348	Enhances EZH2 stability	[141]
Ubiquitination	EZH2	Neuron differentiation	Smurf2	K421	Promotes EZH2 degradation and subsequent PRC2 disassociation	[144]
		CDK5-mediated phosphorylation at T261	β-TRCP (FBXW1)	Unknown	Promotes EZH2 degradation, inhibits PRC2 catalytic activity	[136]
		JAK2-mediated phosphorylation at Y641	FBXW7	Unknown	Promotes EZH2 degradation, inhibits PRC2 catalytic activity	[135]
	SUZ12	PLK1-mediated phosphorylation at S539, S541 and S546	Unknown	Unknown	Promotes SUZ12 degradation and subsequent PRC2 disassociation	[137]
		DZNep treatment	PRAJA1	Unknown	Promotes EZH2, EED and SUZ12 degradation and subsequent PRC2 disassociation	[145]
SUMOylation	EZH2		Unknown	Unknown	Unknown	[150]
	SUZ12		PIASXβ	K75	Unknown, dispensable for PRC2 localization and catalytical activity	[150]
O-GlcNAcylation	EZH2		OGT	S73, S76, S84, S87 and T313	Enhances stability of isolated EZH2	[154, 155]
			OGT	S729	Critical for H3K27me2/3 catalysis	[155]

### Methylation

**Fig. 4:**
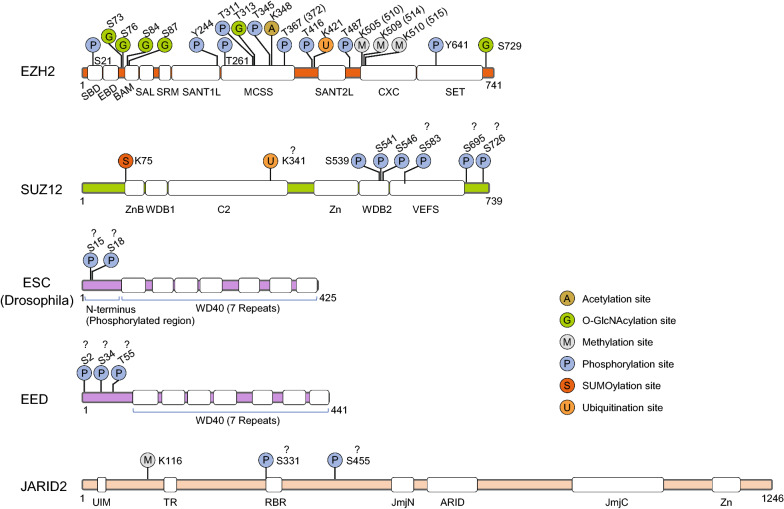
PRC2 subunits are modified by PTMs. The domain architecture of the PRC2 subunits and the schematic mapping of various PTM sites in the protein sequence are shown. PTM sites in EZH2, SUZ12, EED and JARID2 were taken from the indicated publications or public databases in PhosphoSitePlus^®^ (https://www.phosphosite.org) with a minimum of 5 references, while the PTM sites in ESC (*Drosophila*) were predicted from public databases in SCANSITE 4.0 (https://scansite.mit.edu) with the sites matching the predicted motif sites of Casein Kinase 2 (CK2). SBD, SANT1-like binding domain; EBD, EED-binding domain; BAM, β-addition motif; SAL, SET activation loop; SANT1L/2L, SANT1/2-like; MCSS, motif connecting SANT1L and SANT2L; CXC, cysteine-rich domain; SET, catalytic domain of EZH2; ZnB, zinc finger-binding; WDB1/2, WD-40 binding domain1/2; Zn, Zn finger region; VEFS, VRN2-EMF2-FIS2-SUZ12 domain; UIM, ubiquitin-interaction motif; TR, transrepression; RBR, RNA-binding region; JmiN/C, Jumonji N/C; ARID, AT-rich interaction domain

Earlier studies have identified H3K27 as the sole substrate of PRC2. Recent breakthroughs, revealed that PRC2 can not only catalyse the methylation of H3K27 but also methylate many other non-histone proteins, including itself [68, 123, 124]. Indeed, several subunits within PRC2 have been reported to be methylated by PRC2 itself, with different effects on its functions in catalytic activity. The core subunit EZH2 can be automethylated at K510, K514 and K515, residues located in the critical regulatory region adjacent to or within its CXC domains (Fig. 4) [113, 125]. This automatic methylation occurs before the allosteric activation of PRC2, but is dispensable for the recruitment of PRC2 to chromatin. However, the methylation of EZH2 at K510 and K514 is critical for H3K27me3 catalysis, and mutations in these sites disturb the methylation of H3K27 in vitro and in vivo since they can facilitate access of the catalytic pocket of PRC2 to histone H3K27 substrate [113, 125]. In addition to EZH2, the facultative subunit JARID2, as mentioned above, can be methylated by PRC2 at K116, leading to the allosteric activation of PRC2 [100]. Notably, JARID2-K116me3-mediated stimulation might be important for the establishment of Polycomb domains in chromatin, since it can trigger the positive feedback loop of PRC2 before its interaction with H3K27me3 even prior to PRC2 initial recruitment to facilitate de novo deposition of H3K27me3 in chromatin [100]. In addition, another core subunit, SUZ12, can also be methylated [113], and the potential methylation site is located in or adjacent to its C2 domain that binds to AEBP2, since the addition of AEBP2 to PRC2 can improve its enzyme activity but may cause a steric effect that hinders SUZ12 methylation [26, 113]. Whether the methylation of SUZ12 will affect the catalytic activity of PRC2 remains to be further studied.

### Phosphorylation

Phosphorylation is the most common PTM on PRC2 subunits that regulates its catalytic activity and chromatin targeting. Notably, phosphorylation of different sites on the PRC2 subunits by different protein kinases may result in different effects on PRC2 function (Table 1). The phosphorylation of EZH2 at S21 by PKB/Akt (protein kinase B) may inhibit PRC2 catalytic activity by disturbing its interaction with histone H3, leading to a decrease in H3K27me3 and the consequent activation of silenced genes [126]. In contrast, the phosphorylation of EZH2 at T372 by P38α and at T345 and T416 by CDK1/2 (cyclin-dependent kinase 1/2) are critical for PRC2 targeting to specific loci [127–130]. Indeed, phosphorylation at T345 may promote PRC2 interaction with HOTAIR (HOX Transcript Antisense RNA) [127, 128], while phosphorylation at T372 and T416 can enhance PRC2 binding to YY1 (Yin Yang 1) and NIPP1 (Nuclear inhibitor of protein phosphatase-1) [127, 129], respectively, and all of them can mediate or stabilize the binding of PRC2 to chromatin. In addition, several phosphorylation sites in EZH2 may regulate PRC2 assembly; for example, the phosphorylation of EZH2 at T311 by AMPK (AMP-activated protein kinase) [131], Y244 by JAK3 (Janus Kinase 3) [132] and T487 by CDK1 [133] may disrupt its association with EED or SUZ12, leading to decreased methyltransferase activity of PRC2. In addition, the phosphorylation of EZH2 at T345 and T487 by CDK1 [134], at Y641 by JAK2 [135], and at T261 by CDK5 [136] can lead to the subsequent ubiquitination and degradation of EZH2. These observations suggest a role for EZH2 phosphorylation that is linked to ubiquitination and thus leads to degradation. In addition to EZH2, SUZ12 can be phosphorylated by mitotic polo-like-kinase1 (PLK1) at S539, S541 and S546 to regulate its binding to EZH2, and phosphorylation at these sites can also promote the ubiquitin-mediated degradation of SUZ12, indicating that the phosphorylation of SUZ12 also affects the assembly of PRC2 [137]. Finally, phosphorylation of the N-terminus of *Drosophila* ESC (mammalian EED homologue) by CK1/2 (Casein kinases 1/2) results in homodimerization, and it is required for the formation and stability of a larger PRC2 complex containing PCLs and histone deacetylase RPD3 [138].

### Acetylation

Acetylation is a reversible PTM that is regulated by histone acetyltransferases (HATs) and histone deacetylases (HDACs) [139, 140]. Recent breakthroughs have revealed that EZH2 can be acetylated by PCAF (P300/CBP-associated factor) at K348, and this acetylation can be deacetylated by SIRT1 (sirtuin 1) [141]. Acetylation of EZH2 at K348 is dispensable for the assembly of PRC2, nor does it affect PRC2 catalytic activity; however, it functions by inhibiting T345 and T487 phosphorylation to stabilize EZH2 and thus enhances PRC2 capacity in target gene repression [141]. Nevertheless, our understanding of the relationship between acetylation and PRC2 subunits is very limited, and further investigations are urgent to determine whether acetylation in the PRC2 subunits will affect its overall function.

### Ubiquitination and de-ubiquitination

Ubiquitination is a reversible process that regulates protein stability and functional activity by covalently attaching ubiquitin molecules to protein substrates [142, 143]. Several PRC2 subunits have been reported to be regulated by the ubiquitination proteasome system, leading to the degradation of these subunits and disassociation of the PRC2 complex, which indicates that the protein level of the PRC2 subunit can be dynamically regulated through PTMs (Table 1). Notably, although PRC2 can be degraded by ubiquitination, the same PRC2 subunit may be ubiquitinated by different E3 ubiquitin ligases in different contexts, and the mechanism triggering their occurrence may be different. During neuron differentiation, Smad ubiquitination regulatory factor-2 (Smurf2) can mediate the polyubiquitination of EZH2 at lysine 421, thus leading to the ubiquitin‐proteasome‐dependent degradation of EZH2 and subsequent disassociation of PRC2 [144]. Other PTMs (e.g., phosphorylation) may function as decoys or binding sites to induce the ubiquitination of PRC2 subunits in different contexts. As mentioned above, the phosphorylation of EZH2 at Y641 and at T261 can direct E3 ubiquitin ligase β-TRCP/FBXW1- and FBW7-mediated EZH2 ubiquitination, respectively, while mutation in these phosphorylation sites can abrogate EZH2 ubiquitination and stabilize the PRC2 complex [135, 136]. In addition to EZH2, the phosphorylation of SUZ12 at S539, S541 and S546 can also promote its ubiquitination and subsequent degradation during hepatitis B virus-induced liver carcinogenesis, although the corresponding E3 ubiquitin ligases are unclear [137]. In addition, several chemical reagents or inhibitors can also induce the ubiquitination of PRC2 subunits. For example, the histone methylation inhibitor DZNep can induce the ubiquitination of EZH2, SZU12 and EED mediated by the E3 ubiquitin ligase PRAJA1, leading to the rapid degradation of these subunits and the dissociation of the PRC2 complex [145]. Finally, PRC2 subunits can be stabilized by de-ubiquitination in different cell contexts. Ubiquitin-specific protease 21 (USP21) and USP3 can deubiquitinates and stabilize EZH2 and SUZ12, respectively, while the depletion of these proteases results in the degradation of EZH2 and SUZ12 [146, 147]. Collectively, ubiquitination and de-ubiquitination are reversible processes to fine-tune the stability of PRC2 upon the activation of signalling cascades or exert their functions depending on context.

### SUMOylation

Similar to ubiquitination, SUMOylation occurs when a small ubiquitin-like modifier (SUMO) protein is conjugated to the lysine residues of the target protein [148, 149]. In contrast to ubiquitination, which leads to the degradation of the protein substrate, SUMOylation has been reported to affect protein localization, conformation and interactions [148, 149]. Several PRC2 subunits are subjected to SUMOylation both in vivo and in vitro; however, their functional implications for PRC2 activity remain unclear [150]. In particular, SUZ12 can be SUMOylated by the E3 ligase PIASXβ at K75, while SUZ12 K75 SUMOylation is dispensable for PRC2 localization and catalytical activity, and its function is still unclear [150]. It seems that EZH2 can be SUMOylated at multiple sites, since both the in vitro SUMOylation assay and western blot analysis have detected multiple EZH2-modified bands; however, the precise SUMOylation sites of EZH2 and their effects on PRC2 activity remain to be further investigated [150]. Collectively, our understanding of the relationship between SUMOylation and PRC2 is still very limited, and more studies are needed on whether SUMOylation of these PRC2 subunits will affect the overall function of PRC2. Since SUMOylation may regulate protein interaction, especially protein–protein and protein–DNA interaction, it is worth exploring whether SUMOylation occurring on EZH2 will affect the assembly and activity of PRC2 and even the targeting of PRC2 to chromatin.

### O-GlcNAcylation

Protein O-GlcNAcylation is a reversible PTM process that occurs ubiquitously in both the cytosol and nucleus [151, 152]. It can covalently attach β-N-acetyl-D-glucosamine (GlcNAc) moieties to the serine or threonine residues of the target protein by the O-linked N-acetylglucosaminyltransferase (OGT), thereby regulating protein stability, localization, and interaction [151, 152]. Proteomic analysis of the OGT interactomes in HeLa cells revealed that OGT is physically associated with PRC2 subunits, including EZH2, EED and SUZ12, suggesting that the PRC2 core subunits may be potential substrates of OGT in vivo [153]. Indeed, studies have identified several O-glycosylation sites of EZH2, including S73, S76, S84, S87, T313 and S729 (Fig. 4), and their functions have been initially elucidated [154, 155]. Accordingly, EZH2 O-GlcNAcylation does not affect PRC2 assembly but occurs at the N-terminal region, including the S73, S76, S84, S87 and T313 sites, which may regulate the stability of the isolated EZH2, while O-GlcNAcylation of EZH2 at S729 is required for the methyltransferase activity of PRC2 to catalyse H3K27me2/3 [154, 155]. Another study revealed that O-GlcNAcylation of EZH2 affects its binding to the promoter regions of FOXA1/C1 (Forkhead box protein A1/C1), suggesting that EZH2 O-GlcNAcylation might affect the targeting of PRC2 to chromatin [156]. Overall, these observations suggest that O-GlcNAcylation of EZH2 may regulate the catalytic activity and genomic targeting of PRC2. However, the exact mechanism of these actions is still unclear, and whether other PRC2 subunits are modified by O-GlcNAcylation and their functional implications remain to be further studied.

Taken together, these findings support that various PTMs within the PRC2 subunits may ultimately regulate PRC2 activity, including PRC2 stability and assembly, catalytic activity, and even genomic targeting. Different PTMs may have different effects on PRC2: methylation is more likely to regulate the catalytic activity of PRC2, and ubiquitination probably tends to regulate the stability and assembly of PRC2, while the effect of phosphorylation is more complicated. In addition to the abovementioned functions, phosphorylation may also participate in the recruitment of PRC2. Moreover, the same PTM on different PRC2 subunits or even the same PTM at different sites on the same subunit may also lead to different outcomes. As a result, the interplay between different PTMs and PRC2 is complex or comprehensive than simple or simplex, and these PTMs balance each other to modulate PRC2 is currently far from clear. Nevertheless, these PTMs provide multiple processes to fine-tune the functional status of PRC2 in response to different signal cascades and thus regulate PRC2 function depending on context.

## Conclusions

PRC2 is recruited to CGIs to propagate H3K27me3 and maintain transcriptional gene repression, and this process is regulated by multiple factors, particularly cis chromatin features, PRC2 facultative subunits and PTMs of PRC2 subunits. The chromatin features, including DNA sequence and structures characteristics, DNA methylation, and histone modification, determine mRNA transcription statuses and, ultimately, the retention of PRC2. Specific features in the chromatin can be recognized by the PRC2 subunits, while the PTMs on the PRC2 subunits can affect PRC2 stability and assembly, catalytic activity, and even crosstalk with TFs and RNA. In addition, these PTMs provide multiple processes to fine-tune the PRC2 functional statuses in response to different signal cascades. These signals (chromatin features, facultative subunits and PTMs) are likely to work together in a unified model for the precise regulation of PRC2 to fulfil its function in different contexts.

Although much progress has been made regarding the mechanisms regulating PRC2 activity, many questions remain to be resolved, including how PRC2 subunits sense chromatin features, such as the specific sequence and the structure of the DNA motif as well as DNA methylation (Fig. 2). Moreover, it remains unclear how PRC2 balances the core subunits and each facultative subunit in different contexts and how much these balances contribute to its overall function. In addition, high-throughput (HTP) tandem mass spectrometry (MS2) analyses have identified many PTM sites on the PRC2 subunits (data from PhosphoSitePlus^®^) (Fig. 4). However, the corresponding enzymes that catalyse the majority of these PTMs remain to be identified, and their biological significance is largely unknown. Finally, how the PTMs of PRC2 subunits crosstalk to modulate the function of PRC2 and how these PTMs respond to environmental and cellular signal cascades remain unclear. Overall, understanding the precise mechanisms regulating PRC2 activity remains a central and outstanding issue in the field. Addressing these mysteries surrounding PRC2 will provide critical information for us to understand the biological properties of PRC2 in both normal biology and human disease, and shed our lights on the development of novel therapeutic strategies targeting PRC2 activity.

## Supplementary information

**Additional file 1.** A list of known post translational modification (PTM) sites in the human polycomb repressive complex 2.

## Data Availability

Not applicable.

## Abbreviations

PTMs: Post-translational modifications
PcG proteins: Polycomb group proteins
PRC1/2: Polycomb repressive complex 1 and 2
PREs: Polycomb response elements
CGIs: CpG islands
TFs: Transcription factors
EZH1/2: Enhancer of zeste homolog 1/2
EED: Embryonic ectoderm development
SUZ12: Suppressor of zeste 12
RBBP4/7: Retinoblastoma binding protein 4/7
PCLs: Polycomb-like proteins
PHF1: PHD finger protein 1
MTF2: Metal response element binding transcription factor 2
PHF19: PHD finger protein 19
PALI1/2: PRC2-associated LCOR isoform 1/2
EPOP: EloBC and PRC2 associated protein
AEBP2: AE binding protein 2
JARID2: Jumonji and AT-rich interaction domain containing 2
EZHIP: EZH inhibitory protein
EloB/C: Elongins B and C
MLL/COMPASS family: Mixed lineage leukaemia/complex of proteins associated with Set1 family
CDK1/2: Cyclin-dependent kinase 1/2
HOTAIR: HOX Transcript Antisense RNA
YY1: Yin Yang 1
NIPP1: Nuclear inhibitor of protein phosphatase-1
AMPK: AMP-activated protein kinase
JAK2/3: Janus kinase 2/3
CK1/2: Casein kinases 1/2
Smurf2: Smad ubiquitination regulatory factor-2
FBXW1: F-box/WD repeat-containing protein 1
FBW7: F-box and WD repeat domain-containing 7
DZNep: 3-Deazaneplanocin A
